# Factors influencing the experience of sexual and reproductive healthcare for female adolescents with perinatally-acquired HIV: a qualitative case study

**DOI:** 10.1186/s12905-017-0485-9

**Published:** 2017-12-08

**Authors:** Gertrude Mwalabu, Catrin Evans, Sarah Redsell

**Affiliations:** 1 University Lecturer, Kamuzu College of Nursing, University of Malawi, Lilongwe, Malawi; 2 Associate Professor, School of Health Sciences, The University of Nottingham, Nottingham, UK; 3 Professor, Faculty of Health, Social Care and Education, Anglia Ruskin University, Cambridge, UK

**Keywords:** Malawi, Perinatally-acquired HIV, Sexual and reproductive health, Young women

## Abstract

**Background:**

Young people living with perinatally-acquired HIV require age-appropriate support regarding sex and relationships as they progress towards adulthood. HIV affects both genders but evidence suggests that young women are particularly vulnerable to sexual abuse and more prone to engaging in sexual behaviours to meet their daily survival needs. This can result in poor sexual and reproductive health (SRH) outcomes. HIV services in Malawi provide support for young women’s HIV-related clinical needs, but it is unclear whether there is sufficient provision for their SRH needs as they become adults. This paper explores the sex and relationship experiences of young women growing up with perinatally-acquired HIV in order to understand how to improve SRH care and associated outcomes.

**Methods:**

A qualitative case study approach was adopted in which each ‘case’ comprised a young woman (15–19 years) with perinatally acquired HIV, a nominated caregiver and service provider. Participants were purposively selected from three multidisciplinary centres providing specialised paediatric/adolescent HIV care in Malawi. Data was collected for 14 cases through in-depth interviews (i.e. a total of 42 participants) and analysed using within-case and cross-case approaches. The interviews with adolescents were based on an innovative visual method known as ‘my story book’ which encouraged open discussion on sensitive topics.

**Results:**

Young women reported becoming sexually active at an early age for different reasons. Some sought a sense of intimacy, love, acceptance and belonging in these relationships, noting that they lacked this at home and/or within their peer groups. For others, their sexual activity was more functional – related to meeting survival needs. Young women reported having little control over negotiating safer sex or contraception. Their priority was preventing unwanted pregnancies yet several of the sample already had babies, and transfer to antenatal services created major disruptions in their HIV care. In contrast, caregivers and nurses regarded sexual activity from a clinical perspective, fearing onward transmission of HIV and advocating abstinence or condoms where possible. In addition, a cultural silence rooted in dominant religious and traditional norms closed down possibilities for discussion about sexual matters and prevented young women from accessing contraception.

**Conclusion:**

The study has shown how young women, caregivers and service providers have contrasting perspectives and priorities around SRH care. Illumination of these differences highlights a need for service improvement. It is suggested that young women themselves are involved in future service improvement initiatives to encourage the development of culturally and socially acceptable pathways of care.

**Electronic supplementary material:**

The online version of this article (10.1186/s12905-017-0485-9) contains supplementary material, which is available to authorized users.

## Background

Several recent World Health Organisation (WHO) reports highlight that globally, adolescents lack access to adequate health provision [1–3]. These reports note that adolescents living with certain health conditions, such as HIV infection, face particular difficulties in realising their right to healthcare due to its stigmatised nature [1, 4]. Countries around the world are being challenged to assess and improve their services for adolescents [3, 5]. The WHO has produced standards that can be used to drive forward quality improvements in adolescent health. One of these (Standard 8), is that adolescents should be actively involved in decisions about their own care and participate in planning and evaluating services around their needs [3]. A key pre-requisite for service improvement is for healthcare administrators to understand the needs and experiences of adolescents and those who work with them [6]. The challenges of adolescence, particularly around sexual health and relationships are highly gendered and, therefore, it is important to appreciate the different needs and experiences that young women may have from those of young men. This paper reports research findings on the experience of seeking and receiving sexual and reproductive healthcare from the perspective of female perinatally HIV infected adolescents, their primary care givers and health providers in Malawi, a resource poor country in southern Africa.

Worldwide (and in Malawi), an increasing number of children with perinatally-acquired HIV continue to survive into adolescence and beyond. Over 2 million adolescents aged 10–19, and 5 million young people aged 15–24 are living with HIV globally [2]. In Malawi it is estimated that out of an overall population of 15.4 million, almost 1 million people are living with HIV. Approximately 10% of these are children and young people, and over 90% of these have acquired HIV perinatally [7].

As advances in anti-retroviral therapy (ART) have enabled HIV positive young people to grow up and make the transition into adulthood, a key part of their ongoing well-being relates to their sexual and reproductive health (SRH). Many young women in sub-Saharan Africa (including Malawi) initiate sex by the age of 15 or earlier, mostly with older partners, and these encounters are overwhelmingly unprotected [8, 9]. The consequences of unprotected sex include unwanted pregnancies and sexually transmitted infections. For young women living with HIV, this includes potential onward transmission of HIV (to the male partner) and possible re-infection with new strains of HIV [10, 11]. A high level of unwanted pregnancy has been reported among perinatally HIV infected young women living in Malawi [7]. In addition, research shows that young women are particularly vulnerable to sexual abuse [12]. These issues present HIV services with a challenge of how best to meet young women’s SRH needs [13, 14].

Many prominent feminist scholars in Africa have argued that childbearing is central to an African woman’s identity [15]. Malawi is no exception when it comes to the high value placed on motherhood even among women living with HIV. Findings of prior studies conducted in sub-Saharan Africa suggest that the desire to have children, especially since the advent of ART, ranges from 45 to 75% [16, 17]. Several factors have been identified as influencing the women’s desire for pregnancy and childbearing. These include increased self-esteem, maintaining sexual relationships, being pressurised by sexual partners or family members, meeting socio-cultural expectations and as a strategy to create one’s own family particularly in situations of difficulty with intimacy, trust and satisfaction in adult relationships [18, 19]. However, younger age has been documented as a consistent predictor of fertility intentions in all the studies of women living with HIV [20, 21]. In Malawi, this is of clinical significance as many new HIV infections are occurring in younger women as compared to their male counterparts. HIV prevalence is 4.9% among young women and 1% among young men age 15–24 in Malawi [22]. Despite evidence showing that young women living with HIV demonstrate strong desires for childbearing, most programmes do not specifically address fertility desires of this unique group of women [23]. Therefore, service providers’ knowledge of socio-demographic factors motivating young women’s desires for childbearing is imperative for sexual risk reduction counselling and reproductive care.

Research into the experiences of young women growing up with perinatally-acquired HIV is limited, particularly in the sub-Saharan African region. Existing studies have highlighted a range of individual, social, cultural, health-service and structural barriers that negatively influence HIV-infected adolescents’ ability to access healthcare [12, 24]. There is evidence that many young women find it difficult to access contraceptives offered through adult services or by adults - as the area of premarital sexual relations falls into a discursive cultural and social taboo [9, 24]. In addition, evidence suggests that health professionals encounter challenges in addressing young women’s distinctive SRH needs [13, 14]. This is attributed to service providers being ill-equipped to discuss sexual issues with young people [8]. As such, young people point consistently to a need for more information, open communication, and friendly and flexible services [3]. The existing studies in sub-Saharan Africa have drawn upon mixed samples of men and women and have, therefore, been unable to explore gender-specific issues in any detail [12, 24–26]. Our study adds to this body of knowledge by focusing specifically on sexual/reproductive health and on young women’s experiences. In addition, it is the first study on this topic to specifically consider the Malawian context.

## Methods

### Research context

Malawi is characterised by a high incidence of poverty and high HIV prevalence rates among females compared with males [27]. Women in Malawi have little access to formal education and income-generating opportunities [28]. Similarly, many perinatally HIV infected adolescents, have lost one or both parents and are living with foster families, hence are more likely to be living in poverty [29]. In this context, sexual activities are often employed as an economic strategy for modernity or idealised lifestyle, or as means of survival [29]. Nonetheless, the HIV management services are predominantly organised around paediatric and adult care rather than adolescent care. As we argue below, this potentially poses a barrier to young women accessing the services they need and is incongruent with the national sexual and reproductive health and rights policy in Malawi, which advocates contraceptive use for everyone aged 15 years and above [30].

There were three study sites, all located in central Malawi, and all specialist paediatric HIV management centres, Maziko, Chiyembekezo and Yankho (pseudonyms). Maziko Centre is located in an urban area and manages 10% of all children commenced on antiretroviral therapy (ART) in Malawi [7]. Yankho Clinic is a facility affiliated to Maziko Centre situated in a rural setting. Chiyembekezo Centre is the first specialist centre in Malawi for the care and support of people living with HIV including children [31]. There is little specific provision for young people transitioning from child to adult within the health systems of these three Centres. The other services offered in the Centres include counselling and teen club meetings which are conducted once every month. The main activities include: medical care (including condom distribution), group discussions on sexual and reproductive issues and games. The staff involved in the provision of the care include paediatricians, clinical officers, nurses, counsellors, and community volunteers.

### Methodology

An interpretive case study design was adopted. A qualitative case study approach was considered appropriate because it built a comprehensive picture, and yielded in-depth understanding and explanations of the life experiences and needs of young women from different perspectives [32, 33]. Each case comprised of a young woman with perinatally-acquired HIV (15–19 years), her nominated caregiver and service provider. The involvement of caregivers and service providers facilitated a diverse and in-depth understanding of young women’s experiences and enabled young their needs and experiences to be understood in the context of their wider lives and encounters with the health system [34].

### Ethical approval

The study received ethical approval from the University of Nottingham Research and Ethics Committee in United Kingdom in 2011 (C 09 2011) and College of Medicine Research and Ethics Committee in Malawi Review Board in 2011 (P.09/11/1124). Each of the three research sites gave written permission confirming agreement to participate in the study. Young women who became distressed or reported abuse were referred for support services within the centres.

### Access, recruitment and sampling

Participants were recruited by purposive sampling. The inclusion criteria were that young women should: (i) have perinatal exposure to HIV, (ii) be aware of their status, (iii) have attended the Centre for a minimum of 6 months, and, (iv) have the cognitive capacity to complete the data collection techniques. Participants were approached face to face through the clinic. Healthcare professionals at the Centres approached both young women who met the inclusion criteria and their primary caregivers on an individual basis to ask them to participate in the study. The young women and their caregivers who expressed an interest in participating in the study were invited to meet the researcher, who discussed in detail what the study involved. When the researcher had obtained their informed consent, the pair was recruited into the study. Adolescents under age 18 could assent for their participation in tandem with parental permission. The young woman and her caregiver were then given an opportunity to identify a service provider who had been in constant contact with the young woman for at least 6 months. Consent was obtained from the service providers who were interested in participating in the study. Verbal and written consent to take part in the study was obtained from all participants. Participants who were illiterate marked the consent form with a right thumbprint and a healthcare professional signed the form as a witness.

Eight young women were recruited from each HIV management centre, and four from the rural facility, in order to achieve an adequate number of complete cases. However, out of these 20 young women, ultimately only 14 were able to comprise ‘complete cases’ (i.e. each case comprised of a young woman, her caregiver and a service provider). Hence, a total of 42 participants were involved in the study. Four cases were from Maziko Centre, six from Chiyembekezo centre and four from Yankho District Hospital. Thus ten cases were recruited from the urban setting and four from rural location. Six adolescents dropped out because their caregivers were not willing to participate in the study to have complete cases, for fear of being associated with the adolescent’s HIV status. Only young women whose caregivers also participated in the study were given a full explanation about the use of ‘my story’ books and took part in the sentence completion exercise. The participants knew that the researcher had previously worked in the clinic.

This research was conducted with the older age range of female adolescents who attended the Centres (ages 15–19 rather than 10–14). The lead researcher (GM) is female and had previously worked as a professional nurse/midwife at the central hospital which is affiliated and working in partnership with the HIV management centres. At the time of research was pursuing her PhD studies. She felt that this age group would have broader experiences and be more open about sexual issues than younger adolescents. Moreover many young women in sub-Saharan Africa including Malawi initiate sex by the age of 15, mostly with older or non-cohabiting sexual partners for material support and survival, and these encounters are overwhelmingly unprotected [8, 35]. This often results in unplanned pregnancies and early childbearing.

### Data collection

Following an initial period of observation at the three sites in order to develop familiarisation with the settings and contexts, data was collected between January and November 2012. Data was collected for 14 cases (i.e. a total of 42 participants). Data collection involved in-depth individual interviews using a topic guide, and were digitally recorded. All interviews were conducted in Chichewa and were translated and transcribed straight into English by the lead researcher (GM). The majority of participants in the study preferred to have their interviews conducted at the centres than at home. Only four caregivers chose to have their interviews at home. In situations, where interviews were conducted at participants’ homes, other family members were asked to stay at a distance, or sit outside of the house where possible, while the interview was in progress to ensure privacy and confidentiality. The single, open-ended question posed to initiate the interviews with the adolescents, caregivers and service providers was, “can you tell me about your story of growing up with HIV?” Or “can you tell me your experience of caring for or providing care to this adolescent, who is growing up with the virus?” (See Additional file 1, Additional file 2 and Additional file 3). Adolescents were allowed to describe the big-picture issues such as living with HIV, growing up and being a young woman with HIV, and revealed challenges encountered in coping with a positive HIV status. Both caregivers and service providers were encouraged, where possible, to focus their discussions more on the case they were involved with (rather than upon themselves). Further questions included the major needs of a young woman, main issues/challenges of looking after or providing services to a young woman growing up with HIV and most important and realistic strategies in meeting her needs as she grows up to adulthood. Prompts and probes developed as the interviews progressed to encourage the respondents to think more deeply and facilitate openness for the complexity and uniqueness of individual experiences, challenges and perceived needs for young women. Familiarity and closeness with the study settings and ability to engage in regular conversation in the local dialect also assisted the researcher to collect data using some informal interactions that occurred in daily conversation. The researcher’s “insider” perspective facilitated access to more ‘in-group’ activities, such as attending teen club meetings which could not be accessed by an outsider.

The interviews with the adolescents included the use of a technique called ‘my story book’. This is an innovative visual method designed to encourage open discussion on sensitive topics [36] and was particularly useful for participants with low literacy levels. ‘My story’ books comprised of researcher-generated images (depicting different life experiences and events) and sentence completion exercises. Images were culturally sensitive as they depicted young women from a similar ethnic group that the participants could identify with. Young women were invited to put stickers on the images that best suited their different experiences, major needs and issues that impacted upon their lives, future aspirations and priorities. For the sentence completion exercise, they reflected upon the relevance of the chosen images to their experiences, needs and challenges. Upon completion of the “my story” book exercise, adolescents were asked to take part in an in-depth interview during which they were asked to reflect upon what they had written. They were also asked to elaborate on their chosen images and the meaning(s) they attached to them in relation to growing up with HIV. ‘My story’ was pretested at a different facility offering HIV care to determine its feasibility in the Malawian context. Second follow-up interviews were conducted with some of the adolescents regarding issues arising from initial interviews that required clarification, but were not as structured or detailed as the initial interviews. A repeated interview was conducted with one caregiver (aged 21, a sister to an adolescent) who became distressed as she was narrating about the nature of the relationship with their aunt. Since the interview was being conducted within the centre, the distressed participant was referred for support services.

Interviews with the care-givers and service providers followed a topic guide. The researcher made detailed field notes during the data collection phase and the interview transcripts were read contemporaneously to facilitate decision about data saturation and ongoing sampling [37]. Conducting each interview took 30 min to one and half hours.

### Data analysis

Data analysis was a multi-step process involving inductive thematic analysis within and across cases [34, 38] to produce more contextually grounded, transferable findings. A focus on individual accounts within each ‘case’ was important to be able to view each case individually within its own context and remain true to the case study approach [39]. Cross-case analysis was then undertaken to identify similarities, differences, relationships and contradictions. QSR NVivo 10, was used as a tool to systematically identify, sort, code and categorise data for the fine-grained, detailed analysis and comparison of cases [40]. Themes were derived from the data and the researcher (GM) coded the data. To enhance the rigour of the interpretive process, emerging themes were discussed as a team and with selected respondents. Researcher also conducted participant validation exercise through meeting during which all participants commented that the researcher’s interpretation of the data was related to their personal experiences.

## Results

The findings represent 14 complete case studies. The sample of young women, caregivers and service providers were heterogeneous. Six young women were living with one or both biological parent/s (father/mother), two were married and living with their husbands and six lived with either an aunt, an uncle or a sister because both their parents were deceased. Five young women were still attending school (four in secondary school and one in primary school), one was in college, one had completed studies at a tertiary level, two dropped out of school (due to failed exams and lack of fees) and five had children and did not attend school. Five of the caregivers were working, six not working and three were doing business on a small scale. The service providers were from different health backgrounds, including nursing (7), medical (4) and social welfare (3).

(See Tables 1 and 2 for the socio-demographic details of young women, caregivers and service providers).

**Table 1:** Socio-Demographic Details: Young Women

Pseudonym	Age	Setting	Parental status	Caregiver *All aunts (sisters to their mothers*)	Literacy level	Age on ARVs	Age Known status	Teen club	Sexual behaviour					
									*Age of initial relationship*	*Current sexual relationships*	*First sex*	*No. of partners*	*Child bearing age*	*Marital status-married*
Ziliwe	18	Urban	Double orphan	Aunt	Form 4	15	15	√	15	x	15	unknown	17	x
Nane	19	Urban	Maternal orphan	Father	College	12	14	√	15	x	16	2	x	x
Penina	19	Urban	Maternal orphan	Aunt	Form 4	13	13 accidental	√	17	√	x	1	x	x
Ulemu	18	Urban	Double orphan	Sister	Form 3	11	17 accidental	√	x	x	x	x	x	x
Mwatitha	18	Urban	Double orphan	Aunt	Form 3	16	16 accidental	√	12	x	12	3	x	x
Gonjetso	16	Rural	Double orphan	School Headmaster	Form 3	13	13	√	x	x	x	x	x	x
Fatsani	17	Urban	Both alive	Mother	Form 2	13	15	√	14	√	14	4	x	x
Alindine	19	Rural	Double orphan	Aunt	Teacher’ college	13	13	√	17	x	x	1	x	x
Chitsanzo	19	Rural	Double orphan	Sister	Form 2	11	11	x	14	√	15	3	17	√
Tawina	18	Rural	Maternal orphan	Uncle	Form 3	15	15	x	14	√	14	5	x	x
Zaiwo	19	Urban	Paternal orphan	Mother	Std 5	2	8	√	13	x	14	1	14	x
Tamando	17	Urban	Both alive	Husband	Form 1	10	10	√	11	√	12	1	15	√
Dalo	19	Urban	Both alive	Mother	Std 8	12	12	√	12	√	12	unknown	15	√
Tanyada	16	Urban	Both alive	Both (M&F)	Std 8	10	12	√	x	x	x	x	x	x

**Table 2:** Socio-Demographic Details: Caregivers and Service Providers

Category of participants	Characteristics	Number
Caregivers	Age group (years)	
	20–30	4
	31–40	3
	> 40	7
	Relationship to adolescent	
	Mother	3
	Father	2
	Sister	2
	Husband	1
	Aunt	4
	Uncle	1
	School headmaster	1
	Marital status	
	Married	11
	Single	1
	Widow/widower	2
	Literacy level	
	Primary level	2
	Secondary level	10
	Professional	2
	HIV Status	
	Unknown	5
	Negative	1
	Positive	8
	Source of income	
	Not working	6
	Small business scale	3
	Working	5
Service providers	Age range	
	20–30	2
	31–40	8
	> 40	4
	Gender	
	Female	8
	Male	6
	Marital status	
	Married	7
	Single	5
	Widow/widower	2
	Professional status	
	Nurses	7
	Clinical officers	3
	Counsellors	3
	Paediatrician	1

The findings revealed a complex picture of factors that influenced young women’s sexual behaviour and associated health seeking. It also enabled marked differences to be uncovered between caregivers, service providers and young women in terms of the meanings they attributed to sexual relationships and understandings of risk. These issues have been organised into four themes: (i) Wanting to be ‘Normal’; (ii) Risk, health and sex: conflicting agendas; (iii) Cultural silence: responding to adolescent relationships, and, (iv) Getting the care right: health service response.

### Wanting to be ‘normal’

Young women in this study had a strong desire to live as ‘normal’ lives as possible even when faced with multiple challenges associated with their HIV. As such, they sought to behave and look like their non-HIV positive female peers.

There was a strong belief amongst the young women that they were not unwell despite caregivers, peers and service providers often talking about them as ‘sick’. For example, Mwatitha, Tamando and Dalo had downplayed their HIV status and engaged in everyday activities, including multiple sexual relationships from the age of 12 years. They struggled to identify with an image of a young woman living with an illness (HIV). Dalo and her mother illustrate these contrasting perspectives:

*“You wouldn’t want to be who they think you are; I am not sick, I have it (HIV) but am like any other girl. I want to look like them…..at 12 I also started doing it (sex)…….” (Dalo, 19)*

*“My two girls are HIV positive; as they are growing I always thought, they know that they are ill, they cannot do it (sex)……….” (Mrs Mwatipa, Dalo’s caregiver, 38)*

The majority of young women who had lost a parent/s and were living in foster families reported being deprived of basic necessities such as food, clothing, education and financial support. They expressed a longing to be like their peer group and this led some of them to adopt life styles or behaviours that would help them to meet their perceived needs (such as having a boyfriend who could buy them presents), as explained by Ziliwe:

*“…….I wanted to look like them (peers), how could I get nice clothes? Aunt cannot afford, he (boyfriend) provided for all my needs. …..”* (Ziliwe, 18)

Young women also described seeking relationships in order to find love, acceptance and recognition. The majority of them expressed a desire for others to see them as friends, like anyone else - and as worthy of recognition and love. Thus, through their relationships, they sought acknowledgement that being HIV positive was only one part of their personhood.

*“Having a sexual partner is very reassuring that I am like any other girl who can be loved; at least someone has accepted me as I am; I share with him my concerns, discuss issues freely and feel loved; he is very reassuring …….” (Tawina, 18)*

In contrast, there was a strong belief amongst caregivers and service providers that HIV positive young women should be sexually abstinent. Reasons for this were associated with religious/cultural norms and to protect the adolescents’ health (see below). However, the young women found abstinence challenging because having a sexual relationship was a key route to gaining love and acceptance. They noted that the advice on abstinence did not resonate with the reality of their desire for relationships through which they felt they could improve their social status and enhance peer group acceptance and support.

*“We emphasise on abstinence to prevent HIV transmission, but since most girls engage in sexual relationships for money to buy better outfits, they do the same to look attractive like anyone else….” (Mr Zidelu, Fatsani’s service provider, 26)*

*“….my parents could not provide it all, I looked different from others; he provided for me. How could I abstain? I need to look like my friends; I started doing it (sex) at 12 and I looked like them….” (Dalo, 19)*

### Risk, health and sex: conflicting agendas

The research found that the perception and meaning of sexual relationships and potential associated risks was different between the young women, caregivers and service providers. For young women, risk was conceptualised in a social sense – risk of losing a partner, risk of losing material support and the risk of no longer being like everyone else:

*“….he (Mr Mwendo) has been supporting me since I was 10 years ………I felt like paying back in kind (exchanging his kindness with sex); how about transmitting the virus to him? How could I suggest condom use? If he knew my status, I felt like losing my SACCO (Savings and Credit Cooperative – a money lending agency in Malawi); I conceived……he married me.”* (Tamando, 17)

In most interviews, young women talked of pregnancy as the main ‘health-related’ risk of sexual relationships. For them, an unwanted baby clearly posed a financial and social burden and they recognised that it might cause complications with their HIV management.

*“…. He provides for all my needs, but if I become pregnant, will I not be getting sick often; how will I cope with the baby? Will the baby not be an added burden on me? My medication will not be disturbed?” (Nane, 19)*

On the other hand, some of the participants wanted a baby to feel more accepted, to gain social status as a mother and to receive love from their child.

*“Am very excited that I have a baby, my live treasure; am a mother and married despite my HIV status.” (Chitsanzo, 19)*

For young women, avoidance of pregnancy through methods such as condom use carried with it a key social risk associated with the disclosure of their HIV status. For women who had not disclosed their status (the majority of the sample) to their sexual partner, prevailing guidance to use condoms was seen as potentially threatening to a relationship. They believed the use of condoms could not be enacted without a partner finding out about their HIV in the process of negotiations. Hence, most young women assumed that status disclosure and condom use would inevitably lead to rejection or loss of their sources of support. As such, the majority did not consistently use condoms in sexual encounters for fear of jeopardising their goals for the relationship. This resulted in a number of adverse outcomes for the young women including unwanted teenage pregnancies. Zaiwo relates her experience:

*“…….how could I tell him about my HIV status? How could I suggest condom use? Could he not terminate the relationship? It remained a pin code (a secret) for two years. Now I have a baby.”* (Zaiwo, 19).

The young women’s dependence on sexual partners for support meant that male partners assumed power and control in sexual encounters. This inhibited young women’s efforts to negotiate for safer sex, particularly when their partners were much older than them.

*“……being young, they cannot disclose status to negotiate for safer sex with older partners whom they look to for support…. Instead are impregnated…..” (Mrs Khambi, Tamando’s service provider, 53)*

The majority of the young women wanted to be in control of their lives. Many participants noted that, for them, injectable contraceptives would be an ideal solution:

*“…we need to have services we want like injectable contraceptives not condoms. In future I intend to have two children; live my own life, we will be counselled on how to have HIV free children.” (Penina, 19)*

Drawing from Penina’s account, it seemed that she strongly valued child bearing. A striking divergence in viewpoints was where caregivers desired that young women should be given injectable contraceptives to avoid teenage pregnancy or childbearing when they get married. Yet all young women expressed desire to get married and have children. The majority of the young women valued child bearing as a strategy of maintaining their sexual relationships and meeting society’s expectations, hence avoiding societal reproach and promoting their self-worth as young women and as a mark of their femininity. This was evidenced by their subsequent pregnancies and lack of compliance with service providers’ and their caregivers’ instructions on contraceptive use. For instance, after her first abortion, Chitsanzo was very grateful to God that He had made her life purposeful and her womanhood complete by giving her a second child thus promoting her self-worth as a young woman.

*“I lost my first pregnancy but now am very excited that I have a baby, my sexual partner will marry me; am a mother now despite my HIV status; others are unable to bear a child though happily married.”* (Chitsanzo, 19)

However, some expressed frustration in their lack of control because of unequal power relations within their sexual relationships and because of service providers’ reluctance to support this approach, as shown by this quote:

*“…….Injectable contraceptives - not for young ones – they have several effects including delayed fertility return and anaemia, but still there is room for HIV transmission. And how about their health?”* (Mr Mbalame, Ulemu’s service provider, 38)

Caregivers also expressed concern about the consequences and risk of adolescents’ sexual relationships. For many caregivers, their many worry was also about unintended pregnancy (rather than STIs). They saw pregnancy as a severe potential economic risk – an additional burden on family units that were often already over stretched. They also worried about the impact that pregnancy might have on the adolescents’ health and the risk of the baby contracting HIV. For this reason, many care givers disapproved of, and tried to discourage, their adolescents’ sexual relationships:

*“…I don’t want another HIV positive child. Will child bearing not have negative effect upon her health? What if the baby is HIV positive? The baby will be a burden.”* (Ms Ndengu, Penina’s caregiver, 45)

Interviews with the service providers highlighted that many found it difficult to reconcile young women’s sexuality with their HIV status. Their accounts indicated that they felt primarily concerned with the prevention of other STIs and HIV transmission (i.e. health risks associated with sex) rather than with young women’s social identity or economic needs. For this reason, and in order to follow National SRH policy (Ministry of Health, 2009) service providers (and the Centres in general), emphasised condom use. Hence, service providers emphasised abstinence or recommended condom use despite the increased numbers of teenage pregnancies. Alternative forms of contraception were not promoted as these were feared to encourage promiscuity (see below).

*“Most of them are poor and seek financial support from men; but always we advise on abstinence or say to myself should I really give her condoms or not; but she is positive, how about transmitting the virus to her sexual partners; how about the policy? I reluctantly offer condoms after asking lots of questions.” (Mrs Yinde, Alindine’s service provider, 45)*

### Cultural silence: responding to adolescent relationships

Malawi is a religious and socially conservative society and this shaped norms around care. In terms of sexual health, service providers and caregivers were often ambivalent about the advice/service they provided to young women, tending to focus on ideals and morals rather than focusing on practical SRH needs. For example, both caregivers and service providers expressed the views that that those who are not married should abstain from sexual activities. The providers openly recognised that this made them reluctant to offer contraceptives to young women, which was contrary to the Malawi National Sexual and Reproductive Health policy.

*“…. our culture and religious values, ‘no sex till marriage’ you just feel like you are not doing the right thing discussing sex and offering contraceptives to adolescents. So we promote abstinence. ” (Mrs Yinde, Alindine’s service provider, 45)*

Another influence of culture related to the way that provider-patient relationships were often paternalistic, taking on a ‘parent-child’ dynamic. This was particularly the case for adolescents where their service providers were much older and had often known the adolescents for many years. The parent-child dynamic prevented many of the young women from being open about sexual issues with the service providers for fear of being discovered to be sexually active and due to feelings of embarrassment in a context where discussions about sex with elders would be considered to be culturally disrespectful. Service providers also recognised their parental attribution, noting that it made them restrained about providing contraceptives to young women often preferring to emphasise abstinence. They further described a real conflict they found themselves in, between promoting young women’s sexual well-being, following institutional guidance about promoting condoms and facilitating social/religious norms (abstinence).

*“…..In our culture, parents are discouraged to talk about sex with their children; mum or service providers don’t freely talk with me about contraceptive use or sexual issues apart from abstinence or condoms.” (Zaiwo, 19)*

*“….assuming parental role, it’s like you are encouraging your own child to engage in sexual activities. Culturally not acceptable…..” (Mr Nandi, Service Provider, 38)*

These dominant social norms coalesced to create a culture of silence which prevented all parties from openly discussing sexual health issues:

*“….how could you open up on sexual issues when you are quizzed like their child, while others are hearing? Here, it is ‘no to sex’ for unmarried…though in danger, we do it (sex).” (Tawina, 18)*

The young women knew that caregivers and service providers disapproved of their sexual activity so they pretended to conform to social expectations and were not open about sexual relationships. However, their sexual activity did not go unnoticed, as exemplified by Ms. Benga:

*“…..It’s more about their positive status, yet have other needs; we advise them to abstain, but she was also not being honest…” (Ms Benga, Zaiwo’s service provider, 33)*

The consequence was that neither party felt able to be open about sexual issues. This meant that young women would not publicly collect condoms (even though they were available in some Centres). Likewise, the service providers did not want to be perceived as encouraging young women to initiate sexual activities.

*“………when they say, ‘I do not indulge in sexual activities,’ you don’t discuss sexual issues, not even offering condoms? We need youth friendly trainings, we’re often judgemental for they are our children?” (Mr Hanuya, Ziliwe’s service provider, 33)*

### Getting the care right: health service response

In terms of service provision, young women identified several activities organised by the Centres’ teen clubs as being helpful for them. The information on sexual and reproductive issues was described as helping them to understand what was happening to them sexually and physically. However, although group meetings had advantages, there were also disadvantages, the most obvious being that group meetings were not able to address highly personal needs or questions:

*“I benefit from teen club, but I wished sexual issues were discussed in consultation rooms to individuals, to talk freely our challenges…...”* (Gonjetso, 16)

*“…..service providers need to hear our concerns as individuals; he could not use condom, how about his support? I did it (unsafe sex). Only if we discussed possibly these pregnancies could have been prevented.”* (Chitsanzo, 19)

In contrast, service providers’ did not seem to recognise this draw back (or perhaps saw it as a way to avoid difficult encounters). All of them spoke positively of the group meetings and felt they fulfilled an important way of providing sexual and relationship education:

*“Teen club meetings emphasise on behavioural change; in groups they discuss psychosocial affairs, family planning and status disclosure which promote their sexual well-being….”* (Mr Hanuya, Ziliwe’s service provider, 32)

Whilst a close paternalistic relationship posed barriers to discussions about sex, long term bonds were also highly appreciated and were seen to have many advantages in terms of promoting continuity of care. When adolescents become pregnant, they described a fragmented service provision and a lack of continuity as a key problem, leading to further disempowerment:

*“We are seen by different providers during each visit, we don’t continue from where we stopped. We’re completely lost at antenatal clinic, where you are a stranger in a strange environment.”* (Dalo, 19)

Young women who became pregnant often ‘disappeared’ or were discharged from teen club and then referred to antenatal clinic in different health facilities. Service providers highlighted that such lack of continuity of care could contribute to young women’s loss to follow-up in terms of their HIV care.

*“…..due the setup of our clinics and being busy, there is no continuity of care, young women are transferred amongst providers, from HIV centre to antenatal clinic; and are lost to follow up.”* (Mrs Rwinu, Penina’s service provider, 33)

Young women noted that they would prefer a ‘one-stop shop’ type of service that integrates HIV care, SRH and antenatal services. They felt vulnerable and judged within mainstream antenatal services, and also preferred having their own specific support group to share experiences and challenges as young HIV positive mothers.

*“During pregnancy I attended antenatal clinic with adults; who talked about me as if I had committed a crime. We need all the services under one roof, including our own support group…”* (Zaiwo, 19)

## Discussion

This study provides insight into key issues that influence the SRH experiences of young women growing up with perinatally acquired HIV in a sub-Saharan African context. The findings revealed young women’s underlying motivations and desires that led them into sexual relationships. It showed how these relationships could be complex yet important for young women’s wellbeing - both in terms of providing a sense of ‘normal’ self and social identity, providing love and - providing access to important material and social resources. The findings reveal that young women, their care givers and service providers understood these relationships in very different ways, and that these different understandings created a cultural silence acting as a barrier for accessing contraception or for open discussion of other potential health risks. Finally, the study showed how the current structure of service provision, in HIV, SRH and maternity care was not able to meet young women’s needs.

### Rhetoric, reality and consequences for sexual health

The majority of the young women in this study were from low income households. In this context, sexual activities were employed as a way to find love, social acceptance, as an economic strategy for improving their lifestyles, or as means of survival. This exposed them to early marriage and unwanted pregnancies. A growing body of qualitative studies support the findings that many impoverished women and girls engage in such sexual relationships as means of survival [5, 29]. Similarly, several studies have shown that perinatally HIV infected young people engage in sexual activities for pleasure and financial gains [24, 26, 41]. Services need to be oriented to this reality. By contrast, the findings revealed how cultural and religious norms expressed by care givers and service providers shut down possibilities for open recognition of, and discussion of, sexual activities. Young women in Malawi are taught to abstain from sex until they are married [42, 43] and service providers could not reconcile this view with the reality of young women’s lives. Therefore, sexual well-being was medicalised, and, as a result, whilst useful, the teen clubs did not always meet young women’s individual needs. Service providers tended to rely on standard advice to use condoms – in order to protect health – rather than addressing the real issue for young women - which was unwanted pregnancy and social stigma due to disclosure of their HIV status. The study clearly shows that HIV positive young adolescent women need to have access to a wider range of contraceptives, and on-going support to consider how and when to disclose their HIV status in the context of their sexual relationships.

Culturally young women in Malawi are taught to abstain from sex until they are married [42]. Similarly, there is a growing evidence in Africa that service providers and counsellors advise young people living with perinatally acquired HIV to abstain from sexual activities but the young people desire otherwise [24, 44]. The findings in our study show that majority of the young women strongly desire to have children in the future and/or continue having children despite knowledge of the risks associated with childbearing and resistance from others in the community. Furthermore, the findings suggest that young women value child bearing as a mark of womanhood/femininity, to maintain sexual relationships or to meet societal expectations, hence promoting their self-worth and avoiding societal reproach. Culturally in Malawi, childbearing is of utmost importance to both women and men, and signifies a mark of masculinity and femininity [45]. This dominant construction of masculinity and femininity limits the agency of young women living with HIV to demand for their SRH rights like consistent use of condoms. In our study this was evidenced by increased incidence of pregnancies in the centres to which the service providers turned a blind eye. Similar findings were reflected in a qualitative study conducted in South Africa which suggest the need for health professionals and family members to provide non-judgemental support to women living with HIV as the reproductive desires, cultural expectations and experiences of the women are deeply rooted within the community and the family [46]. There is much importance placed on childbearing as a cultural identity marker in most African societies particularly on its centrality to a woman’s identity as a wife, a mother or caregiver including those living with HIV. Therefore childbearing is a positive agency within which young women can exercise power, control and make decisions despite their positive sero-status.

### The conundrum of patient-professional relationships within SRH services

Another common concern amongst the young women in this study was the ‘parent-child’ relationship that they had with service providers. This close relationship hindered communication about sexual issues - a phenomenon that has also been documented in other studies [47, 48]. Similarly, in Malawi, premarital sex and parent-child discussion about sexual issues is culturally forbidden for the fear of influencing young people to initiate sex too early or because sexual discussions are customarily held in secret within families [9, 49]. This prevented the majority of young women from being open about sexual issues with the service providers for fear of being discovered to be sexually active. Our study seems to suggest that the ‘parental’ nature of patient-professional relationships within young people’s HIV care poses a significant conundrum for service development in this area. Our findings indicate that close relationships are indeed very important for maintaining trust and retention in HIV care, yet simultaneously act as barriers to discussions about SRH. The teen clubs were valued and appeared to offer a possible solution, but our study shows that they do not meet individual needs for information or access to non-condom contraception. Any developments in service provision must take this conundrum into account. One way forward would be to involve young women in new service developments and seek their suggestions for appropriate models of care. This kind of approach would be strongly consistent with the recent literature in this area and with WHO Guidelines that emphasises the positive outcomes of empowering and involving young people through participatory approaches to service development [50, 51].

### Study limitations

This study had a number of limitations. All participants were recruited from multidisciplinary Centres where HIV related health services were readily available and service providers had specialised training and experience in the field of HIV and AIDS. Young women who were lost to follow-up and accessing services in peripheral facilities who are likely the most marginalised and most vulnerable to social exclusion and inequalities were not approached because they were harder to reach. The sample excluded young men with perinatally acquired HIV who also need assistance accessing SRH care. Although young women cited numerous instances of male needs, beliefs and behaviours, the findings may not adequately represent the male perspectives. It must be noted that the experiences of the young men could be different or could share some similarities.

### Conclusions and recommendations

The situation described in this study reveals the complexity of trying to implement SRH services for young HIV positive women in a resource constrained and conservative society. It shows a picture that is contrary to Malawi’s National SRH and Rights Policy [30], which advocates contraceptive use for everyone aged 15 years and above, and emphasises young people’s access to SRH services that provide respect and informed consent [30]. It seems that HIV management services in Malawi are currently not effectively prepared to deal with the young women’s complex SRH issues [14].

Some of the issues identified in this study (such as gender inequality or poverty) will be hard to tackle in the short to medium term, important as they are. However, improving information, advice, support and access to contraceptives for young HIV positive women does not require a major new investment of human or material resource. Rather, these are issues that can be tackled through relatively modest service innovations and staff training. New training initiatives should involve young people and should draw upon the results of qualitative studies such as this one, to highlight clearly where differences in values and understandings exist, the consequences they have for service provision and to foster debate and innovation to tackle some of the conundrums.

## Additional files

Additional file 1: Interview guide for the young women. (DOCX 20 kb)

Additional file 2: Interview guide for the caregivers. (DOCX 20 kb)

Additional file 3: Interview guide for the service providers. (DOCX 20 kb)

## Abbreviations

AIDS: Acquired immune deficiency syndrome
ART: Antiretroviral therapy
GM: Gertrude mwalabu, PhD; master of public health; bachelor of science in nursing; university certificate in midwifery
HIV: Human immunodeficiency virus
QSR NVivo: Qualitative data analysis software
SACCO: Savings and credit cooperative
SRH: Sexual reproductive health
STIs: Sexually transmitted infections
WHO: World health organization
