# Supplementary material for: Factors influencing the experience of sexual and reproductive healthcare for female adolescents with perinatally-acquired HIV: a qualitative case study
Source: BMC Womens Health. 2017 Dec 8;17:125. doi: 10.1186/s12905-017-0485-9 (PMC5721479; doi:10.1186/s12905-017-0485-9)
Supplement: Supplementary file 1 — Interview guide for the young women. (DOCX 20 kb) [file 12905_2017_485_MOESM1_ESM.docx]

**INTERVIEW GUIDE FOR ADOLESCENTS (BASED ON SENTENCE COMPLETION EXERCISE)**

**DEFINITIVE SENTENCE COMPLETION EXERCISE IN “MY STORY” BOOK**

Major themes:

- Experiences of growing up with HIV infection
- Major needs/issues that impact on adolescent as they grow up to adult hood and adult care. May include:
- Sexual and reproductive health
- Disclosure of HIV status
- ART adherence
- Psychosocial support
- Future aspirations and priorities.
- Other medical issues

NB: Have a look at the pictures and choose those that best match your responses to the questions to be completed and use the space provided to write your response or create an image that suits your response best.

If you cannot read or write, *the researcher will explain to you how* you should just put stickers on images that best match your responses.

1. **Experiences of growing up with HIV infection (Use yellow stickers for images depicting your responses)**

| **Questions** | **Responses** |
| --- | --- |
| Tell me your story about growing up with HIV (an adolescent will be asked to explain more about the response during interviews).   - What makes you say that? |  |
| How do you feel about having HIV?  (Put stickers on images that depict your responses)  What do you think makes you feel like that? |  |
| How does having HIV effect your:  - relationships with peers  - relationships with family members  - future plans  If the relationship is affected or not affected, what do you think are the possible reasons for that?  How about the reasons for the effects you have mentioned upon your future plans? |  |

1. **Major needs/issues as you grow up to adulthood and adult care (use green stickers for images depicting needs/issues)**

| **Questions** | **Response** |
| --- | --- |
| What might be your major challenges or difficulties of living with HIV as you are growing up to adulthood (Put stickers on images that suit your major challenges or create images).  What are your reasons for choosing these images? |  |
| Who do you like to talk to about your condition? (Put stickers on pictures that suit your preferred individuals).  Why talking to the people chosen?  Why not talking to the others about your condition? |  |
| What do you hope for in the future? (Put stickers on pictures that suit your future desires or create images).  Do you think your condition influenced you in any way in regard to images chosen?  If so how?  If not, why not? |  |
| What helps you to cope up with living with your condition? (Put stickers on pictures that depict on issues that help you to cope or create images)  How does that help you cope up with your condition? |  |
| What do you think are the most important and realistic strategies in meeting your needs?  Why do you think so? |  |

**TRANSLATED DEFINITIVE SENTENCE COMPLETION EXERCISE - MAFUNSO MU BUKHU LA “NKHANI YANGA”**

**Mitu yake:**

- Zomwe ndikudutsamo kapena kumana nazo pamene ndikukula ndi kachilombo ka HIV
- Zofunika pa moyo wanga pamene ndikukula. Zina mwaizo zikhonza kukhala ngati izi:
- Kuuzidwa kuti ndili ndi kachilombo
- Kumwa mankhwala a ma ARV moyo wanga wonse
- Za kugonana ndi uchembere wabwino
- Chithandizo cha mmaganizo
- Zokhumba zanga zokhudza tsogolo la moyo wanga.

NB:

Mukabokosimo mulembemo yankho lanu kapena mujambule chithunzi chomwe chikufotokoza bwino yankho lanu kapena gwiritsani ntchito mapepala mwapatsidwawo kuyankha mafunsowo ngakhale kujambula zithunzi zoyenera mayankho anu.

Ngati simungathe kulemba kapena kuwerenga *opanga kafukufukuyu akufotokozerani bwino lomwe* kuti mungomata timapepala mwapatsidwato pa zithunzi chomwe zikufotokoza bwino mayankho anu.

1. **Zodutsamo kapena zokumana nazo pamene ndikukula ndi HIV (Matani timapepala ta mtundu wa chikasu pa zithunzi zomwe zikufotokoza bwino mayankho anu)**

| **Mafunso** | **Fotokozani yankho lanu munsimu** |
| --- | --- |
| Ungandifotokozereko nkhani yako yakukula ndi kachilombo ka HIV. (nthawi yokambirana mtsikana adzfunsidwa kufotokoza mwatsatane-tsatane) |  |
| Umamva bwanji kuti uli HIV?  (Matani mapepala kapena jambulani zithunzi zomwe zikufotokoza mayankho anu).  Ndipo chimakupangitsa ndi chiyani kumva chomwecho? |  |
| Kuti uli ndi HIV zimakhudza bwanji:  - ubale wako ndi anzako  - ubale wako ndi a chibale ku nyumba  - tsogolo la moyo wako  Ukuganiza kuti ndi chifukwa chiyani zikukhudza chomwecho? |  |

1. **Zofunika kwambiri kapena zokhudza kweni-kweni moyo wanga ngati mtsikana pamene ndikukula ndi HIV (Matani timapepala tobiliwira pa zithunzi zomwe zikufotokoza bwino mayankho anu)**

| **Mafunso** | **Fotokozani yankho lanu munsimu** |
| --- | --- |
| Ukuganiza kuti zovuta kapena zokhudza kweni-kweni pa moyo wako pamene wokukula ndi kachilombo ka HIV ndi ziti? Chifukwa chiyani?  (Matani mapepala kapena jambulani zithunzi zomwe zikufotokoza bwino mayankho anu). |  |
| Umakonda kuyankhula ndindani zokhuza matenda ako? (Matani mapepala kapena jambulani zithunzi zomwe zikufotokoza bwino mayankho anu).  Chifukwa chiyani iwowo osati ena? |  |
| Ukuyembekezera zotani mtsogolo muno pa moyo wako? (matani mapepala kapena jambulani zithunzi zomwe zikufotokoza zoyembekezera/zokhumba za moyo wanu mtsogolo muno) |  |
| Ukuwona kuti ndi chiyani chomwe chikumakuthandiza pamene ukukula ndi matendawa? (matani mapepala kapena jambulani zithunzi zikusonyeza zinthu zomwe zimakuthandiza) |  |
| Nanga njira zothandiza kwambiri pa zofunika pa moyo wako pamene ukukulamu ndi ziti? (ungafotokozereko mmene zingathandizire) |  |
