# Supplementary material for: Factors influencing the experience of sexual and reproductive healthcare for female adolescents with perinatally-acquired HIV: a qualitative case study
Source: BMC Womens Health. 2017 Dec 8;17:125. doi: 10.1186/s12905-017-0485-9 (PMC5721479; doi:10.1186/s12905-017-0485-9)
Supplement: Supplementary file 2 — Interview guide for the caregivers. (DOCX 20 kb) [file 12905_2017_485_MOESM2_ESM.docx]

**INTERVIEW GUIDE** **FOR CAREGIVERS**

**Introduction**

*Thank you very much for agreeing to participate in this study. As I had discussed with you earlier, I am interested in exploring your experiences of raising up a young woman growing with the illness. I will be asking you to look back and identify her needs as she is growing up to adulthood with the illness. You don’t have to answer all of the questions. Only answer what you feel comfortable with. Anything you say will be completely confidential and will be anonymously reported.*

*Do you have any questions about the interview or the research?*

**Demographic information**

| Gender |  |
| --- | --- |
| Years you have been caring for the adolescent |  |
| Parental status |  |
| Education background |  |
| Residential area |  |
| Marital Status |  |
| Date of interview |  |

**Experiences and challenges of caregivers / Needs of the female adolescents**

1. Can you tell me what it is like to look after an adolescent who is growing up with the illness?

- Can you expand on your response
- What do you think about it?
- Do you have any worries and anxieties about raising up a young woman growing up with HIV?
- If yes, can you tell me about your worries and anxieties?
- How do you plan to handle your worries/anxieties?
- If no, why not…......
- How comfortable/confident do you feel about discussing issues related to your adolescent who is growing up with the HIV with the:
- Adolescent herself
- Which issues? Why? *(probe for each issue)*
- At what age? Why? *(probe for each issue)*
- Health providers?
- Family members?
- Your friends?
- If not comfortable, which issues and why not? *(probe for each issue why not)*

1. What are the main issues/challenges of looking after a young woman growing up with HIV? *(ask the caregiver to explain for each)*

- What have you done so far about them? (*ask the reason for each action*)
- If you have not done anything about the issues, why?
- How do you plan to handle the issues/challenges that you have not done anything about?
- How have the issues/challenges influenced your:
- care for your adolescent
- attitudes towards the experiences and needs of your adolescent
- relationship with adolescent
- relationship with other family members, friends and the community.
- Please share with me what people in the community do to or say about female children/girls growing up with HIV?

1. In your views what would you say are the major needs/issues that affect your adolescent as she is growing up to adulthood and adult care? (*probe even for those that are health realated)*

- Can you describe any particular need/issue that affects your adolescent that you have found it difficult to deal with?
- What do you think are the consequences?

1. How do you support your adolescent regarding living with HIV and SRH issues? *(probe more on what is done and its effect on the adolescent)*

- Who has so far provided assistance in regard to this?
- Can you tell me the type of the assistance provided?
- What do you think could have been done better and how?

1. Which strategies do you think are the most important and realistic in meeting the needs of your adolescent as she grows up to adulthood and adult care?

Is there anything else you would like to add on your views about female adolescents growing up to adulthood in general?

**Concluding remarks**

Thank you so much for sharing your experiences with me. If you have any further queries do feel free to get in touch with me through the number I have provided.

**TRANSLATED INTERVIEW GUIDE** **FOR CAREGIVERS**

**MAFUNSO KWA OSAMALIRA ATSIKANA**

**Poyambira**

*Zikomo kwambiri chifukwa chakuvomera kutengapo mbali mukafukufukuyu. Monga ndinakambira kale ndikufuna kumva kwa inu zomwe mwakumana nazo pamene mukulera mtsikana amene akukula ndi HIV.Ndikufunsani kuti mukumbukire mmene mwakhalira naye komanso kuona zofunika pamoyo wake pamene akukula ndi matendawa. Simukuyenera kuyankha mafunso onse koma okhawo omwe mukuona kuti mukhonza kuyankha. Zonse zomwe tikambirane zisungidwa mwachinsinsi ndipo sizisonyeza kuti munanena ndinu.*

*Kaya muli ndi mafunso pa zomwe tikuti tikambiranezi kapena pa kafukufukuyu?*

**Mbiri yanu**

| Amuna/akazi |  |
| --- | --- |
| Zaka zomwe mwakhala mukumusamalira mtsikanayu |  |
| Ubale wanu ndi mtsikanayu |  |
| Maphunziro munafika pati |  |
| Komwe mukukhala |  |
| Muli pa banja |  |
| Tsiku lofunsidwa |  |

**Zomwe mwadutsamo/zovuta ngati wosamalira mtsikana ndi zofunika pa moyo wa mtsikana wanu**

1. Mungandiuze mmene mumamvera kulera mtsikana yemwe akukula ndi HIV?

- Mungafotokozeko mwatsatane-tsatane
- Mukuganiza kuti ndi chifukwa chiyani mumamva chomwecho?
- Muli ndi madandaulo kapena nkhawa ina iliyonse pamene mukulera mtsikanayu?
- Ngati muli nazo, mungandifotokozereko?
- Ndiye mukuganiza kuti mukhonza kuchitapo bwanji kuti muthetse nkhawazo ndi madandaulo anu?
- Ngati mulibe nkhawa kapena madandaulo mukuganiza kuti ndi chifukwa chiyani?
- Ndinu womasuka bwanji kukamba zokhudza mtsikana wanu amene akukula ndi HIV ndi:
- Mtsikanayo mwini wake
- Monga ngati ziti? Chifukwa chiyani? *(funsani kuti afotokoze pa china chili chonse)*
- Ali ndi zaka zingati? Chifukwa chiyani? *(fufuzani pa china chilli chonse chomwe angakambe).*
- Amene amapeleka chithandizo kwa mtsikanayu –achipatala?
- A pa banja lanu?
- Anzanu wocheza nawo?
- Ngati simuli omasuka kukamba, pa zinthu monga ziti ndipo chifukwa chiyani *(funsani kuti afotokoze pa china chilichonse)*

1. Ndi zovuta zanji zomwe mukukumana nazo pamene mukuyang’anira mtsikanayu amene tsopano akukula ndi HIV?

- Fotokozani mwatsatane-tsane zomwe mukukumana nazo.
- Ndiye mwachitapo chiyani? (Funsani chifukwa chake pa china chilichonse chomwe achita?
- Ngati simunachitepo kanthu, chifukwa chake ndi chiyani?
- Ndipo mukuganiza kuti mupanga bwanji ndi zomwe simunachitepo kanthuzo?
- Zovuta kapena zomwe mukukumana nazo zakhudza bwanji:
- Chisamaliro chanu pa mtsikanayu
- Malingaliro/maganizo anu pa zomwe mtsikanayu akudutsamo ndi zofunika pa moyo wake.
- Ubale wanu ndi mtsikanayu
- Ubale wanu ndi a pa banja panu, anzanu and anthu ena koma inu mukukhalako.
- Mungandiuzeko zomwe anthu amawachita kapena kuwanena atsikana omwe akukula ndi HIV mdera lanu lino?

1. Nanga inuyo mukawuona moyo wa mtsikana wanu, mukuganiza kuti zomwe zili zofunika kapena zomukhudza kwambiri mmene akukulamu ndi zinthu ngati ziti? *(fufuzani zomwe zikukhudzana ndi ku chithandizo cha kuchipatala)*

- Mungafotokozeko mwa zina zokhudza kapena zofunika pa moyo wa mtsikana wanu zomwe mwaziona kuti ndi zovuta kuti mumuthandize?
- Ndipo mukuganiza kuti zotsatira zake zingakhale ziti?

1. Nanga inu ngati womusamalira mtsikanayu, mukumuthandiza bwanji pa nkhani:

- Yokula ali ndi HIV
- Zakugonana ndi uchembere wa bwino *(fufuzani zomwe amachita ndi zotsatira zake pa mtsikana wawo)*
- Alipo wina aliyense amene wakuthandizani kuthandiza mtsikana wanu pa nkhani zoterezi?
- Mungandifotokozereko kuti amuthandiza munjira yanji?
- Mukanakonda zikanachitika mwanjira yanji kuti mtsikana wanu apindule koposa?

1. Mukuganiza kuti njira zoyenera ndi zofunika kwambiri kuthandiza kukwaniritsa zofunika pa moyo wa mtsikanayu pamene akukula ndi ziti?

Pali china chilichonse chomwe mukufuna kuwonjezera zokhudzana ndi chithandizo choyenera achitsikanawa pamene akukula ndi HIV?

**Mau omaliza**

Zikomo kwambiri chifukwa chakundifotokozera zomwe mukudutsamo pamene mukusamalira mtsikanayu. Ngati mungakhale ndi mafunso, khalani omasuka kundipeza kapena kukamba nane pa nambala mwapatsidwayo.
