# Supplementary material for: Factors influencing the experience of sexual and reproductive healthcare for female adolescents with perinatally-acquired HIV: a qualitative case study
Source: BMC Womens Health. 2017 Dec 8;17:125. doi: 10.1186/s12905-017-0485-9 (PMC5721479; doi:10.1186/s12905-017-0485-9)
Supplement: Supplementary file 3 — Interview guide for the service providers. (DOCX 20 kb) [file 12905_2017_485_MOESM3_ESM.docx]

**INTERVIEW GUIDE** **FOR SERVICE PROVIDERS**

**Introduction**

*Thank you very much for agreeing to participate in this study. As I had discussed with you earlier, I am interested in exploring your experiences in providing care to female adolescents growing up with HIV. I will be asking you to reflect back at your experiences and interactions with the adolescent and identify her needs as she is growing up to adulthood with the HIV. You don’t have to answer all of the questions. Only answer what you feel comfortable with. Anything you say will be completely confidential and will be anonymously reported.*

*Do you have any questions about the interview or the research?*

**Demographic information**

| Gender |  |
| --- | --- |
| Professional status |  |
| Working experience:   - in HIV management - with HIV infected adolescents - in adolescent health particularly SRH services. |  |
| Period you been in contact with the adolescent |  |
| Any relevant adolescent health training |  |
| Residential area |  |
| Marital status |  |
| Date of interview |  |

**Experiences and challenges of service providers / Needs of the female adolescents**

1 Can you tell me what it is like to provide care to an adolescent growing up with HIV?

- Can you expand on your response
- What do you think about it?
- Do you have any worries and anxieties about providing care to a female adolescent growing up with HIV?
- If yes, can you tell me about your worries and anxieties?
- How do you handle your worries/anxieties?
- If no, why not…......
- How comfortable/confident do you feel about discussing issues related to their status as they grow up with the HIV with the:
- Adolescent herself – which issues and at what age? Why? *(probe on each issue).*
- Fellow health providers?
- The guardian/parent?
- If not confortable/confident, specify which issues and why not?

1. What are the main issues/challenges of providing care to an adolescent growing up with HIV infection?

- What have you done so far about them as service providers?
- How does this HIV management centre plan to handle the issues/challenges that you have not done anything about?
- How have the issues/challenges influenced the:
- Care provided to female adolescents in this centre?
- Attitudes of staff towards the experiences and needs of these adolescents?
- Relationship of staff and the adolescents?
- Relationship of staff and adolescents’ guardians/parents
- Please share with me what people in the community do to or say about female adolescents growing up with HIV?

1. In your views what would you say are the major needs/issues that affect female adolescents as they are growing up to adulthood and adult care?

- Can you describe needs/issues that affect female adolescents that you have found it difficult to deal with?
- What do you think are the consequences?

1. How do you support female adolescents regarding living with HIV and SRH issues? *(probe more on what is done and its effect on the adolescent)*

- What do you think could have been done better and how?

1. Which strategies do you think are the most important and realistic in meeting the needs of your adolescent as she grows up to adulthood and adult care?

Is there anything else you would like to add on your views about female adolescents growing up to adulthood in general?

**Concluding remarks**

Thank you so much for sharing your experiences with me. If you have any further queries do feel free to get in touch with me through the number I have provided.**TRANSLATED INTERVIEW GUIDE** **FOR SERVICE PROVIDERS**

**Poyambira**

*Zikomo kwambiri chifukwa chakuvomera kutengapo mbali mukafukufukuyu. Monga ndinakambira kale ndikufuna kumva kwa inu zomwe mwakhala mukukumana nazo pamene mukupeleka chithandizo kwa atsikana amene akukula ndi HIV.Ndikufunsani kuti mukumbukire mmene mwakhalira naye komanso kuona zomwe mwakhala mukukamba naye ndi zofunika pamoyo wake pamene akukula ndi HIV. Simukuyenera kuyankha mafunso onse koma okhawo omwe mukuona kuti mukhonza kuyankha. Zonse zomwe tikambirane zisungidwa mwachinsinsi ndipo sizisonyeza kuti munanena ndinu.*

*Kaya muli ndi mafunso pa zomwe tikuti tikambiranezi kapena pa kafukufukuyu?*

**Mbiri yanu**

| Amuna/akazi |  |
| --- | --- |
| Udindo wanu ku ntchito kuno |  |
| Zaka zomwe mwapeleka chisamaliro kwa:   - amene ali ndi HIV - achinyamata (young people) amene ali ndi HIV - achinyamata zokhudzana ndi kugonana ndi uchembere wabwino. |  |
| Nthawi yomwe mwakhala mukupereka chithandizo kwa mtsikanayu |  |
| Maphunziro omwe munapanga wokhudzana ndi za achinyamata |  |
| Komwe mukukhala |  |
| Muli pa banja |  |
| Tsiku lofunsidwa |  |

**Zomwe mwadutsamo/zovuta ngati wopeleka chithandizo kwa mtsikana amene akukula ndi HIV ndi zofunika pa moyo wake**

1. Mungandiuze mmene mumamvera kupereka chithandizo kwa mtsikana yemwe akukula ndi HIV?

- Mungafotokozeko mwatsatane-tsatane
- Mukuganiza kuti ndi chifukwa chiyani mumamva chomwecho?
- Muli ndi madandaulo kapena nkhawa ina iliyonse pamene mwakhala mukupeleka chithandizo kwa mtsikanayu pamene akukula?
- Ngati muli nazo, mungandifotokozereko?
- Ndiye mukuganiza kuti mukhonza kuchitapo chiyani kuti muthetse nkhawazo ndi madandaulo anu?
- Ngati mulibe nkhawa kapena madandaulo, mukuganiza kuti ndi chifukwa chiyani?
- Ndinu womasuka bwanji kukamba zokhudza mtsikanayu mmene alili kuti akukula ndi HIV ndi:
- Mtsikanayo mwini wake
- Monga ngati ziti? Chifukwa chiyani? *(funsani kuti afotokoze pa china chili chonse)*
- Ali ndi zaka zingati? Chifukwa chiyani? *(fufuzani kuti pa chilichonse chomwe angakambe)*
- Anzanu achipatala?
- Amene akumusamalira?
- Ngati simuli omasuka kukamba, ndi zinthu monga ziti ndipo chifukwa chiyani? *(funsani kuti afotokoze pa china chilichonse)*

1. Ndi zovuta zanji zomwe mukukumana nazo pamene mukupeleka chithandizo kwa mtsikanayu amene tsopano akukula ndi HIV?

- Fotokozani mwatsatane-tsane zomwe mukukumana nazo.
- Ndiye mwachitapo chiyani pa zomwe mukukumana nazozo inuyo ngati achipatala? (*Funsani chifukwa chake pa china chilichonse chomwe achita?*
- Ngati simunachitepo kanthu, chifukwa chake ndi chiyani?
- Ndipo mukuganiza kuti mupanga bwanji ndi zomwe simunachitepo kanthuzo?
- Zovuta kapena zomwe mukukumana nazo zakhudza bwanji:
- Chithandizo chomwe mtsikanayu akulandira pa chipatala pano?
- Malingaliro/maganizo anu pa zomwe mtsikanayu akudutsamo ndi zofunika pa moyo wake.
- Ubale wanu inuyo ngati womuthandiza ndi mtsikanayu
- Ubale wanu ndi amene akumusamalira mtsikanayu
- Mungandiuzeko zomwe anthu amawachita kapena kuwanena atsikana omwe akukula ndi HIV mdera lanu lino?

1. Nanga inuyo mukawuona moyo wa mtsikanayu, mukuganiza kuti zomwe zili zofunika kapena zomukhudza kwambiri mmene akukulamu ndi zinthu ngati ziti? *(fotokozani zomwe zikukhudzana ndi chithandizo cha kuchipatala)*

- Mungafotokozeko mwa zina zokhudza kapena zofunika pa moyo wa mtsikanayu zomwe mwaziona kuti ndi zovuta kuti mumuthandize?
- Ndipo mukuganiza kuti zotsatira zake zingakhale ziti?

1. Nanga inu ngati wothandiza mtsikanayu kuno ku chipatala, mukumuthandiza bwanji pa nkhani:

- Yokula ali ndi HIV
- Zakugonana ndi uchembere wa bwino *(fufuzani zomwe amachita ndi zotsatira zake pa mtsikanayu)*
- Mukuona kuti pamayenera kuchitika zotani kuti mtsikanayu apindule koposa kapena athandizike bwino?

1. Mukuganiza kuti njira zoyenera ndi zofunika kwambiri kuthandiza kukwaniritsa zofunika pa moyo wa mtsikanayu pamene akukula ndi ziti?

Pali china chilichonse chomwe mukufuna kuwonjezera zokhudzana ndi chithandizo choyenera achitsikanawa pamene akukula ndi HIV?

**Mau omaliza**

Zikomo kwambiri chifukwa chakundifotokozera zomwe mukudutsamo pamene mukuperka chithandizo kwa mtsikanayu. Ngati mungakhale ndi mafunso, khalani omasuka kundipeza kapena kukamba nane pa nambala mwapatsidwayo.
